# The relationship between food security, fruit and vegetable consumption, and health-related factors in the late COVID-19 pandemic in Czechia: a cross-sectional study

**DOI:** 10.1186/s12889-025-23154-9

**Published:** 2025-07-02

**Authors:** M. Ohno, J. Vávra, P. Jehlička

**Affiliations:** 1 Department of Social Geography and Regional Development, Faculty of Science, Charles University, Albertov 6, 128 43 Prague, Czechia; 2 Department of Local and Regional Studies, Institute of Sociology, The Czech Academy of Sciences, Jilská 1, 110 00 Prague, Czechia

**Keywords:** Food insecurity, Fruit and vegetable consumption, COVID-19, Home food production, Social determinants, Sociodemographic, Socioeconomic, Obesity, Czechia

## Abstract

**Background:**

Food insecurity is one of the social determinants of health and affects dietary quality and well-being. This study aimed to examine the associations among food insecurity, sociodemographic and economic factors, and health-diet characteristics, with a particular focus on fresh fruit and vegetable (FV) consumption during the late COVID-19 pandemic in Czechia.

**Methods:**

Data from a cross-sectional survey, ‘*Living through the Pandemic',* collected in October 2022 as part of a Czech longitudinal survey, were analysed. The study included a representative sample of Czech adults (N = 1,499, aged 20 years and above). Binary logistic regression was performed to assess associations among food insecurity, sociodemographic-economic factors and health-diet factors. Food insecurity was assessed as experiencing or worrying about a lack of food. Sociodemographic-economic factors included sex, age, education, income, number of children and home food production. Health-diet factors included BMI, limited mobility and daily fresh FV intake, defined as eating fresh FV at least once per day. Determinants of daily fresh FV intake were analysed separately.

**Results:**

Over 30% of respondents (*N* = 486) were at risk of food insecurity. Individuals aged 20–34 years, those with lower educational attainment, and those with limited mobility were more likely to report food insecurity. Compared with the high-income category, individuals in the lowest income category had a sevenfold higher likelihood of reporting food insecurity. Food-insecure individuals had approximately twofold higher odds of not having fresh FV daily. The odds of not having fresh FV were particularly higher among younger adults (20–34 years) and males. Individuals with BMI ≥ 25 kg/m^2^ had 30% higher odds of not having daily FV, with marginal significance (*p* = 0.05). Educational attainment, rather than income, was a key predictor of FV consumption. Home food production contributed to better food security and higher FV consumption.

**Conclusion:**

In Czechia food insecurity and the limited FV intake relate to younger adults, socioeconomically disadvantaged individuals, and those with limited mobility. Lower education attainment, rather than income, predicts limited consumption of FV, underscoring the long-term impact of early education on healthy eating. Given the high prevalence of overweight and obesity, inadequate FV intake presents a public health concern. Policies should aim to improve access to affordable and nutritious foods, and strengthen education on healthy eating habits to mitigate long-term health disparities.

## Introduction

The COVID-19 pandemic (hereinafter the pandemic) impacted disproportionately our lives across different sociodemographic and economic groups. The pandemic imposed challenges on various aspects of food security and impacted vulnerable populations such as children, women and elderly populations as well as people from lower socioeconomic groups [1]. Food security refers to conditions in which all people always have access to, both physically and economically, safe and nutritious food to meet individuals’ dietary needs for a healthy and active life [2]. Food security has four dimensions that need to be fulfilled simultaneously and consistently: physical availability of food, economic and physical accessibility, utilization (implying ways in which an individual can absorb and metabolize nutrients, i.e., skills, knowledge, and diversification of diets) and stability [2]. Multiple factors influenced food security during the pandemic: increased unemployment rates, inability to work due to COVID infections, comorbidities, changes in food supply chains and increases in food and energy prices. According to the Food and Agriculture Organization (FAO) Global Food Price Index, the global food price index during the pandemic (2020–2022) rose by 28% compared with that in the three years preceding the pandemic [3]. The food inflation rate during the pandemic in Czechia was one of the highest among the European Union (EU) member states, at 5.6% higher than the EU average inflation rate [4]. A state-wide, population-level survey in the U.S. showed an increase of over 30% in the prevalence of food insecure people during the lockdown compared with the year before the pandemic, and food-insecure individuals expressed physical and economic obstacles to obtaining food [5]. Studies revealed differences in the prevalence of food insecurity based on age and gender during the pandemic. Younger individuals and women were more susceptible to experiencing food insecurity [6–8]. Income levels influence what people can afford to buy and what people choose to eat. Research has shown that low-income households have higher risks of food insecurity with limited income to afford to buy healthy food [9, 10].

The consequences of food insecurity include poor diet quality or malnutrition, poor health, obesity and mental health problems such as anxiety and depression [11–16]. A systematic review that examined the relationship between food insecurity and dietary quality among adults and children in the U.S. revealed that greater food insecurity was associated with lower consumption of fruit and vegetables [17]. Low income and food insecurity were associated with reduced consumption of fresh produce such as fruit and vegetables (FV) that are more nutritious than processed foods. Energy-dense, processed foods high in fat and added sugars are more affordable than fresh FV [18]. Increased intake of energy-dense foods and reduced consumption of FV may have contributed to a higher prevalence of obesity among individuals experiencing food insecurity [13, 19, 20]. Daily consumption of FV has been shown to lower mortality and reduce chronic diseases, certain cancers and obesity [21, 22], and daily consumption of FV is recommended by the WHO [23]. According to the European Health Interview Survey (EHIS) in 2019, 48% of individuals aged 15 and older in Czechia did not consume FV daily [24]. However, the relationship between food insecurity and FV consumption in Czechia remains insufficiently explored. Based on prior studies, we hypothesized that younger individuals, females, and those with lower income would be more likely to experience food insecurity. Additionally, food-insecure individuals would have lower consumption of fresh fruits and vegetables.

The aims of our study are 1) to assess food security and FV consumption among the adult Czech population, using a representative panel from a longitudinal survey; and 2) to identify sociodemographic, economic and health determinants of food insecurity and fruit and vegetable consumption.

## Methods

### Study design and data collection

Data on a representative panel of Czech adults from Wave 44 ‘*Living through the Pandemic*', collected between October and November 2022, as part of a Czech longitudinal panel survey [25], were analysed. The aim of the survey was to investigate health, behavioural changes, mental health and economic impacts on Czech people during the pandemic. Respondents were selected from the Czech National Panel, a pool of respondents willing to participate in market research and public opinion polls. Quota sampling was used to select respondents with these quotas: sex, age, education, municipality size and region. The survey was conducted online (CAWI), and the data were collected by the company NMS Market Research in collaboration with the company PAQ Research and the Systemic Risk Institute (SYRI) consortium. Post-stratification weighting was applied to adjust the overrepresentation of respondents from cities above 50,000 inhabitants in the sample of the collected data. The weighted data represented the Czech population in terms of sex, age, education and place of residence (municipality size and region). Of the 1,699 respondents, those with missing income information (n = 164) and implausible extreme Body Mass Index (BMI) (n = 4) were excluded, resulting in a final analytical sample of 1,531 participants (unweighted). Weighted estimates were applied to ensure population representativeness.

### Measures

#### Assessment of food security

As the primary outcome, one’s food security status was assessed using the following questions: *‘Did it happen to you in the last month that there was not enough food in your household due to a lack of funds to buy it?'* and *‘In the last month, were you worried that there would not be enough food in your household due to a lack of funds to buy it?'.* Those who responded ‘Often' or ‘Sometimes' to either or both questions were categorized as being at risk of food insecurity. Those who chose *‘Never'* were categorized as no risk of food insecurity. These questions were based on the Hunger Vital Sign™, two-question screening which has been validated, to identify individuals at risk for food insecurity [26, 27]. The explanatory variables including seven sociodemographic-economic variables (sex, age, education level, having children, income, municipality size, home food production) and three health-diet variables (Body Mass Index [BMI], mobility and daily FV consumption) were included in our binary logistic regression analyses. These explanatory variables were selected based on previous studies that investigated risk factors for food insecurity and determinants of health inequalities [20, 28]. Age was categorized into four groups: 20–34 years, 35–49 years, 50–64 years, and ≥ 65 years. Education level was based on self-reported highest level of education and categorized into three groups: Low (Primary + lower-secondary education), Medium (Higher-secondary education) and High (tertiary – college or university). For the binary logistic regression analyses, the number of children was dichotomized as having at least one child and no children in the household. Income was assessed based on the equivalized net monthly household income and categorized into three categories: ‘Below the poverty line’ (below 60% of median), ‘Low’ (below median) and ‘Above standard’ (median and higher). Municipalities were grouped into three population size categories: Small (< 5,000), Medium (< 100,000) and Large (≥ 100,000). This classification reflects the nature of the settlements, ranging from rural or suburban areas to mid-sized towns and cities, and finally to large cities, as commonly used in the Czech context. Respondents were also asked to answer whether they produced any food, typically in the form of gardening (yes or no). BMI (weight in kilograms divided by height in metres squared) was calculated using self-reported height and weight values and categorized using the WHO classification: underweight (< 18.5 kg/m^2^), healthy weight (18.5–24.9 kg/m^2^), overweight (25–29.9 kg/m^2^) and obese (≥ 30.0 kg/m^2^) [29]. Owing to a small number of respondents with underweight (1.6%), underweight and healthy weight were grouped together in our analyses. Implausible extreme BMI values (BMI < 10 kg/m^2^, BMI > 80 kg/m^2^) were considered outliers and excluded from our analyses. To measure respondents’ general health (self-perceived health), respondents were asked *‘How is your health in general?'* and were asked to select one answer from the following options: 1. Very good; 2. Good; 3. Fair; 4. Bad; and 5. Very bad. This question addresses one’s self-perceived health, reflects multiple health dimensions [30], and is a standardized question used by the WHO [31]. In our analyses, the responses were dichotomized as good (Answers 1 and 2) and not good (Answers 3–5). To assess how a respondent’s mobility impacted food security status, the respondents were asked *‘Have you been limited in activities that people usually do because of a health problem?'* and were asked to select one of the following options: 1. Yes, severely limited; 2. Yes, limited, but not severely; and 3. Not limited at all. Responses were dichotomized as being limited (Answers 1 and 2) and not limited (Answer 3). This question is derived from the Global Activity Limitation Indicator (GALI), which is a validated single question, to measure longstanding mobility limitations due to health problems [32] and is used in EHIS (HS3 variable) [33]

### Assessment of fruit and vegetable consumption

To understand the impacts of one’s food insecurity status on healthy eating habits, participants were asked to answer questions associated with the frequency of fresh FV consumption. The frequency questions in our study were adapted from validated frequency questions, The 2017 Behavioral Risk Factor Surveillance System (BRFSS) Fruit and Vegetable Module used by the Behavioral Risk Factor Surveillance System developed by the Centers for Disease Control and Prevention (CDC) in the U.S. [34, 35]. The participants were asked *‘How often do you consume the following food categories?—Fresh fruits and vegetables'*. The participants were then asked to select one of the following answers: Rarely or not at all (maximum once per month); 2 to 3 times a month; Once a week; 2 to 3 times a week; 4 to 6 times a week; and Daily. In our binary logistic regression analysis, respondents who reported NOT having fresh FV daily were coded as 1 while respondents who reported having fresh FV daily were coded as 0.

### Statistical analyses

Descriptive statistics were used to summarize the baseline sociodemographic, economic and health-characteristics of respondents on the basis of their food security status and FV consumption during the pandemic. Differences in the baseline characteristics of respondents who reported food insecurity and those who did not were compared using the Chi-squared test for categorical variables and the t-test for continuous variables (age and BMI). Differences in the characteristics of respondents who consumed FV daily and those who did not were also tested using the Chi-squared test for categorical variables and the t-test for continuous variables (age and BMI). Continuous variables are presented as means and standard deviation (SD), and categorical variables are expressed as percentages. Binary logistic regression was performed to assess whether experiencing food insecurity was associated with sociodemographic factors (sex, age, education level, having children in the household, income, size of municipality, and home food production). Respondents who reported being food insecure were coded as 1, and respondents who did not report being food insecure were coded as 0. In Model 1, odds ratios (ORs) with 95% confidence intervals (95% CI) were adjusted for sex (Reference = Male), age (Reference = age 65 +), education level (Reference = High) and having children in the household (Reference = No). Model 2 was further adjusted for equivalized income (Reference = Above standard), the size of the municipality (Reference = Large) and home food production (Reference = Yes). The odds of being food insecure were estimated separately for health-diet covariates (BMI, mobility and daily FV consumption) as prior literature considered health-related variables as determinants of food insecurity, not additional confounders [20]. ‘Limited mobility’, instead of ‘self-perceived health’, was included as a predictor variable for assessing food insecurity due to its strong association with self-perceived health. It directly impacts food access and is a more relevant factor in this context. ORs were adjusted for BMI (continuous), mobility (Reference = Not limited) and daily FV consumption (Reference = Daily). Furthermore, we investigated factors that contributed to not having fresh FV daily. Binary logistic regression was performed to estimate the odds of not having fresh FV. Model 1 was adjusted for food security status (Reference = Not food insecure), age (Reference = Age 65 +), sex (Reference = Female), having children in the household (Reference = No), BMI (Reference = Healthy + underweight) and education level (Reference = High). Model 2 was further adjusted for income (Reference = Above standard), self-perceived health (Reference = Good) and home food production (Reference = Yes). A *p*-value of less than 0.05 was considered significant. Statistical analyses were performed using IBM SPSS, Version 28.

## Results

### Characteristics of the study sample by food security

Characteristics of the weighted study population by food security status are presented in Table 1. The final analytical sample included 1499 individuals, of which 49.1% were men and 50.9% were women. The mean age of the analytical sample was 51.8 years old (SD = 16.4 years). Approximately one-third of the analytical sample was at risk of food insecurity. Statistically significant differences between the food-secure and food-insecure groups were observed for all sociodemographic variables. The analytical sample with food insecurity had a higher proportion of females than those without food insecurity. The proportion of the youngest age group (20–34 years old) with food insecurity was greater than that of those without food insecurity. Nearly 60% of the analytical sample with food insecurity had low educational attainment as compared to those without food insecurity. More than one-third of the analytical sample with food insecurity had an equivalized net monthly household income below the poverty line compared with 11.1% among those without food insecurity. Only 5.8% of the food-insecure group reported the use of food banks.

**Table 1:** Weighted characteristics of respondents by food security status

	**Food secure**			**Food Insecure**			
Unweighted sample	N = 1111	72.6%		N = 420	27.4%		
Weighted	N = 1013	67.6%		N = 486	32.4%		
**Sociodemographic—economic characteristics**	**%**	**95%**	**CI**	**%**	**95%**	**CI**	***p*-value**
**Sex**							
Male	51.4	48.4,	54.5	44.1	39.7,	48.5	0.008
Female	48.6	45.5,	51.6	55.9	51.3,	60.1	
**Age, years (Continuous)**	**Mean**	**SD**		**Mean**	**SD**		
	52.8	16.5		49.5	16.2		<.001
**Age groups**							
20–34	15.8	13.6,	18.1	23.0	19.5,	26.9	0.004
35–49	28.0	25.2,	30.8	27.3	23.4,	31.2	
50–64	25.1	22.5,	27.8	24.4	20.8,	28.5	
65 +	31.2	28.4,	34.1	25.3	21.6,	29.3	
**Having children**							
None	74.9	72.1,	77.4	67.9	63.7,	71.9	0.004
1 or more	25.1	22.5,	27.8	32.1	28.1,	36.3	
**Education**							
Low	40.8	37.8,	43.8	57.7	53.2,	62.0	<.001
Medium	34.3	31.5,	37.3	32.3	28.3,	36.6	
High	24.8	22.3,	27.6	10.0	7.6,	13.0	
**Municipality size**							
Small	32.9	30.0,	35.8	38.9	34.6,	43.3	0.004
Medium	39.3	36.3,	42.3	41.0	36.6,	45.4	
Large	27.8	25.1,	30.7	20.1	16.8,	23.9	
**Equivalized net monthly household income**							
Below poverty (below 60% of median)	11.1	9.3,	13.2	35.5	31.2,	39.7	< 0.0001
Low (below median)	48.1	45.1,	51.3	48.2	43.7,	52.6	
Above-standard (up to 1,5 × of median)	30.7	13.6,	33.6	14.1	11.1,	17.3	
High (more than 1,5 × median)	10.1	25.2,	12.0	2.3	1.2,	3.9	
**Use of food bank**							
Yes	0.1	0,	0.5	5.8	3.9,	8.1	< 0.001
No	99.9	99.5,	100	94.2	91.9,	96.1	
**Home food production**							
Yes	57.9	54.9,	61.0	49.3	45.0,	53.8	0.002
No	42.1	39.0,	45.1	50.7	46.2	55.0	
**Health-diet Characteristics**	**Mean**	**SD**		**Mean**	**SD**		***p*-value**
**BMI (continuous)**							
	28.1	5.8		28.3	7.1		0.626
**BMI**							
Underweight & healthy	32.0	29.2,	34.9	35.2	31.0,	39.5	0.375
Overweight	36.5	33.6,	39.5	33.3	29.3,	37.6	
Obese	31.5	28.7,	34.4	31.5	27.5,	35.7	
**Self-rated health**							
Good	57.4	54.4,	60.5	44.4	40.1,	48.9	< 0.001
Poor	42.6	39.5,	45.6	55.6	51.1	59.9	
**Limited mobility**							
Yes	37.5	34.6,	40.5	48.5	44.1,	53.0	< 0.001
No	62.5	59.5,	65.4	51.5	47.0,	55.9	
**Daily FV consumption**							
Less than daily	69.1	66.2,	71.9	83.4	80.0,	86.6	< 0.001
Daily	30.9	28.1,	33.8	16.6	13.4,	20.0	

Chi-square test was used to compare differences between categorical variables, and independent-samples t-test was used for continuous variables (age and BMI)

More than half the analytical sample with food insecurity reported poor self-rated health compared to the food secure group). Nearly half of the analytical sample with food insecurity reported limited mobility, compared with 37.5% in the food secure sample. The mean BMI did not significantly differ between food-insecure individuals (28.3 kg/m^2^) and food-secure individuals (BMI 28.1 kg/m^2^). More than 80% of food-insecure individuals did not consume fresh FV daily. A greater proportion of those without food insecurity reported growing food than those experiencing food insecurity.

### Assessment of food security with selected determinants

In the weighted logistic regression models shown in Table 2, after controlling for sociodemographic variables, including sex, age, education and living with at least one child in Model 1, being female had higher odds of experiencing food insecurity than being male (OR 1.28, 95% CI 1.02–1.61). Individuals with lower educational attainment had greater odds of reporting food insecurity (OR 4.12, 95% CI 2.88–5.88) than individuals with high educational attainment. Being a young person aged between 20 and 34 years, compared to adults aged 65 years and older, had greater odds of reporting food insecurity (OR 1.96, 95% CI 1.38–2.79). Living with at least one child compared with no children had greater odds of reporting food insecurity (OR 1.61, 95% CI 1.20–2.16). In the fully adjusted model (Model 2), extended by three additional covariates, i.e., equivalized household income, size of the municipality and home food production, odds ratios related to being female were no longer statistically significant. The odds of food insecurity increased for the youngest group (OR 2.53, 95% CI 1.73–3.71), and the second oldest group (aged 50–64 years) appeared as a significant predictor of reporting food insecurity (OR 1.63, 95% CI 1.17–2.28). Although educational attainment remained a significant predictor, this additional control reduced the odds ratios for low educational attainment by 42% and for medium education by 22%. Income level was a significant predictor for reporting food insecurity. Individuals whose income was below the poverty line (OR 7.04, 95% CI 4.83–10.26) and low-income individuals (OR 2.54, 95% CI 1.86–3.46) had higher odds of reporting food insecurity compared to those with income above standard. Individuals without home grown food had higher odds of reporting food insecurity (OR 1.61, 95% CI 1.26–2.06) compared to those who grew food. In the fully-adjusted model, the size of the municipality and living with children were not significant predictors. Table 3 shows associations between food security and health-diet characteristics. Individuals with limited mobility were associated with higher odds of food insecurity compared with those without limited mobility (OR 1.60, 95% CI 1.28–2.01). Individuals who did not consume fresh FV daily had greater odds of reporting food insecurity (OR 2.27, 95% CI 1.73–2.99).

**Table 2:** Adjusted ORs for food insecurity by sociodemographic-economic characteristics

	**Model 1†**			**Model 2‡**		
	**OR**	**95% CI**		**OR**	**95% CI**	
**Sex (male, Ref)**						
Female	1.28*	1.02,	1.61	1.00	0.79,	1.28
**Age (65+, Ref)**						
20–34	1.96**	1.38,	2.79	2.53**	1.73,	3.71
35–49	1.05	0.74,	1.50	1.40	0.96,	2.04
50–64	1.22	0.89,	1.67	1.63*	1.17,	2.28
**Education (High, Ref)**						
Low	4.12**	2.88,	5.88	2.39**	1.64,	3.50
Medium	2.49**	1.72,	3.59	1.94**	1.32,	2.84
**Living with children (No, Ref)**	1.61*	1.20,	2.16	1.34	0.97,	1.84
**Equivalized household income (Above standard, Ref)**						
Below poverty				7.04**	4.83,	10.26
Low				2.54**	1.86,	3.46
**Municipality size (Large, Ref)**						
Small				1.20	0.86,	1.67
Medium				1.12	0.82,	1.53
**Home food production (Yes, Ref)**						
No				1.61**	1.26,	2.06

*p<0.05. **p<0.001

†Model 1: Adjusted for sex, age and education

‡Model 2: Adjusted for sex, age, education, living with children, equivalized household income, municipality size and home-grown food

**Table 3:** Adjusted ORs for food insecurity by health-diet related characteristics

	**OR**	**95% CI**	
BMI (continuous)	1.00	0.98,	1.01
Limited mobility (No, Ref)	1.60**	1.28,	2.01
Daily FV consumption (Daily, Ref)	2.27**	1.73,	2.99

^*^p < 0.05. **p < 0.001

### Characteristics of the study sample by fruit and vegetable consumption

Table 4 shows the characteristics of the analytical sample stratified into two groups on the basis of daily fresh FV consumption. Most of the overall analytical sample did not consume fresh FV daily. Nearly 70% of those who ate FV daily were females, whereas, more than half of those who did not eat FV daily were males. A greater proportion of older adults aged 50 years and above consumed FV daily compared with those who did not consume FV daily. The youngest age group (20–34 years) who consumed FV daily accounted for only 10%. Those who consumed FV daily had a higher proportion of high educational attainment than those who did not consume FV daily (27.8% vs 17.3%).

**Table 4:** Weighted characteristics of respondents by daily FV intake

	**Not having fresh FV daily**			**Having fresh FV daily**			
**Unweighted sample**	N = 1084	70.8%		N = 447	29.2%		
**Weighted**	N = 1106	73.8%		N = 393	26.2%		
	**%**	**95%**	**CI**	**%**	**95%**	**CI**	***p*-value**
**Sex**							
Male	54.9	51.9,	57.8	32.8	28.3,	37.6	< 0.001
Female	45.1	42.2,	48.1	67.2	62.4,	71.7	
**Age (Continuous)**	**Mean**	**SD**		**Mean**	**SD**		
	51	17		55	15		<.001
**Age groups**							
20–34	20.9	18.6,	23.4	10.4	7.7,	13.7	< 0.001
35–49	29.0	26.4,	31.8	24.2	20.1,	28.6	
50–64	21.9	19.6,	24.5	33.1	28.6,	37.8	
65 +	28.2	25.6,	30.9	32.3	27.8,	37.1	
**Number of children**							
None	71.0	68.2,	73.6	77.3	73.0,	81.3	0.015
1 or more	29.0	26.4,	31.8	22.7	18.7,	27.0	
**Education**							
Low	47.2	44.3,	50.1	43.8	38.9,	48.7	<.001
Medium	35.5	32.8,	38.4	28.4	24.2,	33.1	
High	17.3	15.1,	19.6	27.8	23.5,	32.3	
**Size of City**							
Small	34.3	31.5,	37.1	36.5	32.0,	41.5	0.097
Medium	41.4	38.5,	44.3	35.4	30.8,	40.2	
Large	24.3	21.9,	26.9	28.0	23.7,	32.6	
**Equivalized net monthly household income**							
Below poverty (below 60% of median)	20.1	17.8,	22.5	16.1	12.7,	19.9	0.030
Low income (below median)	48.7	45.7,	51.6	46.7	41.7,	51.5	
Above-standard (up to 1,5 × of median)	24.7	22.2,	27.3	26.9	22.8,	31.5	
High (more than 1,5 × median)	6.6	5.2,	8.2	10.3	7.7,	13.7	
**Food insecure**							
No	63.3	60.4,	66.1	79.5	75.5,	83.4	<.001
Yes	36.7	33.9,	39.6	20.5	16.6,	24.5	
**Home food Production**							
Yes	52.1	49.1,	55.0	63.7	58.8,	68.3	<.001
No	47.9	45.0,	50.9	36.3	31.7,	41.2	
**Use of food bank**							
Yes	2.4	1.7,	3.5	0.6	0.1,	1.6	0.017
No	97.6	96.5,	98.3	99.4	98.4,	99.9	
**BMI (continuous)**	**Mean**	**SD**		**Mean**	**SD**		
	28.4	6.2		27.7	6.3		0.741
**BMI**							
Underweight & healthy	31.1	28.4,	33.9	38.4	33.7,	43.3	0.025
Overweight	36.9	34.1,	39.8	31.6	27.1,	36.3	
Obese	32.0	29.3,	34.8	30.0	25.7,	34.7	
**Self-rated health**							
Good	50.9	47.9,	53.8	59.8	54.9,	64.6	0.002
Poor	49.1	46.2,	52.0	40.2	35.4,	45.1	
**Limited mobility**							
Yes	41.2	38.3,	44.1	40.7	35.9,	45.6	0.883
No	58.8	55.9,	61.7	59.3	54.4,	64.1	

Chi-square test was used to compare differences between categorical variables, and independent-samples t-test was used for continuous variables (age and BMI)

### Assessment of consumption of fruit and vegetables with selected determinants

Table 5 shows the results from the weighted binary logistic regression models to investigate factors contributing to not eating fresh FV at least once a day. In Model 1, adjusted for food insecurity, sex, age, educational attainment and BMI, males had more than twice the odds of not eating FV daily compared to females (OR 2.62, 95% CI 2.03–3.39). Those at risk of food insecurity had greater odds of not eating FV daily (OR 2.21, 95% CI 1.65–2.96). The youngest age group (20–34 years) was less likely to eat FV daily (OR 2.51, 95% CI 1.64–3.83) compared to other age groups (Reference category: Age 65 +). Individuals with BMI greater than or equal to 25 kg/m^2^ (overweight or obese) had greater odds of not having FV daily compared to those with BMI ≤ 24.9 kg/m^2^ (OR 1.40, 95% CI 1.08–1.83). The odds of not having FV daily were higher in those with low education (OR 1.90, 95% CI 1.38–2.62) and medium education (OR 2.17, 95% CI 1.54–3.04) compared to those with high educational attainment.

**Table 5:** Adjusted ORs for NOT having fruit and vegetables daily

	**Model 1**†			**Model 2‡**		
	**OR**	**95% CI**		**OR**	**95% CI**	
**Food insecure (NO, Ref)**						
Food Insecure	2.21**	1.65,	2.96	1.89**	1.39,	2.58
**Sex (Female, Ref)**						
Male	2.62**	2.03,	3.39	2.78**	2.13,	3.63
**Age groups (65 +, Ref)**						
20–34	2.51**	1.64,	3.83	3.47**	2.22,	5.44
35–49	1.42*	1.03,	1.97	1.90**	1.34,	2.69
50–64	0.74	0.54,	1.01	0.83	0.60,	1.14
**BMI (Healthy&underweight, Ref)**						
Overweight&obese	1.40*	1.08,	1.83	1.31 (*P* =.05)	1.00,	1.71
**Education, 3 categories (High, Ref)**						
Low	1.90**	1.38,	2.62	1.65*	1.18,	2.31
Medium	2.17**	1.54,	3.04	2.11**	1.49,	2.98
**Income (Above standard, Ref)**						
Below poverty				1.24	0.81,	1.88
Low				1.23	0.91,	1.67
**Self-perceived health (Good, Ref)**						
Poor				1.79**	1.35,	2.37
**Home food production (Yes, Ref)**						
No				1.49*	1.17,	1.96

^*^p < 0.05. **p < 0.001

^†^Model 1: Adjusted for food insecurity, age, sex, BMI and education

^‡^Model 2: Adjusted for food insecurity, age, sex, BMI, education, income, self-perceived health and home food production

In Model 2, after simultaneously controlling for equivalized household income, the size of the municipality, self-rated health, and home food production in addition to covariates in Model 1, food insecurity remained a significant predictor for not eating FV daily (OR 1.89, 95% CI 1.39–2.58). The odds of not having FV daily in the youngest age group increased by 38% (OR 3.47, 95% CI 2.22–5.44). The odds of not having FV daily for the second youngest group (35–49 years) were greater (OR 1.90, 95% CI 1.34–2.69) than the age group 65 +. Being male remained a significant predictor for not eating FV daily, with a slight increase in the odds ratio (OR 2.78, 95% CI 2.13–3.63). Compared with high educational attainment, lower educational attainment remained a significant predictor for not having FV daily. Poor self-rated health was associated with higher odds of not eating FV daily (OR 1.79, 95% CI 1.35–2.37). Lack of home food production was associated with increased odds of not eating FV (OR 1.49, 95% CI 1.17–1.96). The individuals with BMI ≥ 25 kg/m^2^ had higher odds of not having FV daily (OR 1.31, 95% CI 1.00–1.71) compared to those with BMI ≤ 24.9 kg/m^2^, with marginally significant results. Given that the confidence interval includes 1.00, this finding should be interpreted with caution.

## Discussion

To our knowledge, the present study is the first study in Czechia to examine food security in relation to sociodemographic, economic and health-diet factors during the pandemic using a representative sample of respondents. We explored associations between food security and social determinants of a healthy diet. Our analysis revealed that one-third of our sample was at risk of food insecurity during the late pandemic due to financial or resource constraints. In our study, food insecurity was more prevalent among young adults than other age groups, and young adults remained significantly associated with food insecurity after controlling for other sociodemographic and economic covariates. Susceptibility to food insecurity among young adults may be explained by higher unemployment and lower income during the pandemic. In the U.K., young adults under 25 years old were more than twice as likely to work in job sectors that were considered ‘nonessential’ such as hospitality and non-food retailers subjected to shutdown during the pandemic [36] while the suspension of hiring also reduced job opportunities for young adults starting a career or seeking employment [37]. As food insecurity experienced during young adulthood has been shown to increase the incidence of diabetes in later adulthood [38], urgent interventions are needed to address food insecurity among young adults to mitigate the long-term health consequences.

We found that after adjusting for additional sociodemographic covariates in the fully adjusted model, the odds ratio for being food insecure for females ceased to be statistically significant. This suggests that additional socioeconomic covariates (household income, municipality size and home food production) moderated the susceptibility of females to food insecurity. Globally, women experience food insecurity more than men, and gender disparities in education and income are recognized as contributing determinants to the gender disparities in the prevalence of food insecurity [6, 8, 39]. Mane et al. [40] estimated at least 57% reduction in the gender disparity in food insecurity when income, employment and education were equal. This highlights the importance of addressing income inequality as part of strategies to reduce food insecurity. Dudek and Myszkowska-Ryciak [41] similarly found that in Central-Eastern Europe, women were more likely to experience mild food insecurity than men. However, their study also emphasized that socioeconomic factors such as lower education, unemployment, and lower income were also significant contributors to food insecurity, reinforcing our finding that socioeconomic factors moderate the gender disparity. The similarities between our results and those of Dudek and Myszkowska-Ryciak suggest that gendered patterns of food insecurity in Czechia align with neighbouring regional trends.

Consistent with other studies [10, 42, 43], unsurprisingly, adults with incomes below the poverty line exhibited much higher odds of experiencing food insecurity than those with low income or above. Individuals with lower levels of educational attainment also had higher odds of experiencing food insecurity. Educational attainment showed a consistent association with food security after controlling for additional covariates. Among the multitude of factors influencing food security, income and education are widely recognized as major drivers of food insecurity. This was also true in Czechia. With rising food prices, inflation and unemployment, low-income households face greater difficulty affording an adequate and nutritious diet. Low-income households allocate a greater proportion of their income to housing expenses, thereby reducing the amount available for food expenditures [9]. Educational attainment has been identified as one of the social determinants of health, and less education is associated with lower income and poorer health [44, 45]. Our findings suggest an intertwined association between educational attainment and income in relation to the prevalence of food insecurity. One effective public health strategy to reduce food insecurity in Czechia could be implementing a pricing intervention similar to Norway’s approach. Norway’s largest grocery chain introduced a nationwide discount on fruit and vegetables, making fresh produce more affordable for consumers. Research showed that between 2012 and 2020, such pricing strategies successfully increased the purchase and consumption of FV in counties with lower socioeconomic status and higher obesity prevalence [46].

### Health and food insecurity

The association between food insecurity and BMI ≥ 25 kg/m^2^ was marginally significant in the fully-adjusted model. In our study, both groups exhibited similar BMIs, averaging 28 kg/m^2^, which falls within the upper threshold of the overweight category (29.9 kg/m^2^). More than 60% of individuals were classified as overweight or obese in our study. Given that Eurostat reported in 2019 that over 60% of adults in Czechia were either overweight or obese, with nearly 70% of men being either overweight or obese [47], our findings align with these national statistics and suggest that overweight and obesity are prevalent regardless of food security status.

Consistent with earlier studies [48–50], our results showed that adults with limited mobility were more likely to experience food insecurity. Individuals with disabilities face challenges in two pillars of food security: access and utilization of food. Individuals with disabilities may live in poverty and have physical or financial difficulty accessing food and/or preparation (utilization) of food. Our findings confirm emerging evidence that individuals vulnerable to food insecurity often have lower levels of education, limited income and limited mobility, facing elevated health risks. Being food insecure has adverse consequences. Food insecurity has been shown to be associated with poor diet quality [49, 51, 52]. A lower consumption of fresh FV was evident in food-insecure households [17, 53–55] whereas a higher consumption of energy-dense, high caloric, high-sugar diets was associated with food insecurity [9, 56]. Consistent with these earlier studies, low intake of fresh FV was observed more frequently among individuals at risk of food insecurity and among those classified as overweight and obese. Increased daily consumption of FV has been shown to be inversely associated with weight gain [57]. Further research into the consumption patterns of other food items in relation to BMI could provide valuable insights into the current high prevalence of overweight and obesity in Czechia.

The benefits of FV consumption including lowering the risk of cardiovascular diseases and certain cancers have been well established in epidemiological studies [57, 58]. It is alarming that over 70% of the study population in our study did not consume fresh FV daily. The reported number is higher than the earlier finding of Smutná et al. [59] who reported approximately 65% according to the data collected in 2021. While previous studies suggested low income as a predictor of limited FV consumption [16, 60, 61], our analysis indicated that younger age and education level emerged as significant predictors of this lack of FV consumption even after controlling for income. This highlights the critical role of education in shaping eating habits, suggesting that nutrition education at an earlier age may be particularly effective in promoting healthier eating behaviours throughout the life course. In 2020, the Czech Ministry of Health launched the public health strategy ‘*Zdraví 2030’* which outlines a framework for reducing chronic diseases in Czechia through preventive measures including promoting physical activity and health literacy [62]. While the strategy does not specifically include nutrition education, integrating such education into school curricula could foster healthier eating habits in the long term.

We further demonstrated that home food production could have positive impacts on food insecurity and increase the consumption of FV. Our analysis indicated that respondents who did not engage in home food production were associated with experiencing food insecurity and not consuming fresh FV daily. Home, allotment or community gardens influence all four pillars of food security: availability, access, utilization and stability. During the pandemic, despite the disruption of the food supply chain, people who grew food at home or in community gardens were less food insecure [63]. Home food production enhances the ‘access’ and ‘availability’ of fresh FV and alleviates food scarcity in times of food supply chain disruption (‘stability’). Furthermore, gardening has been shown to augment the intake of fresh produce [64, 65], thereby enhancing the diversification of nutrients in the diet (‘utilisation’). In our study, even after controlling for other covariates, gardeners were more likely to be food secure and consume a greater amount of FV. Given the widespread popularity of gardening in Czechia, access to gardens is a highly relevant aspect of public health policies in Czechia.

### Limitation

The present study has some limitations. The study was based on a self-reported survey, and information on health status, the level of limited mobility and height and weight to calculate BMI were not confirmed by official health authorities. This introduces the possibility of reporting bias. The present study is cross-sectional, and therefore, we cannot determine the causal relationship between food insecurity and low intake of fresh fruits and vegetables.

Although food security has four dimensions that need to be satisfied simultaneously at all times, the two-item screening questions did not fully capture the ‘access’ to healthy food and ‘utilization’, the ability to maximise nutritional intake to meet individuals’ dietary needs. As a result, some individuals classified as food secure may still lack access to nutritious food and, therefore, may satisfy their hunger with energy-dense, unhealthy food. Due to a lack of food literacy or the cooking skills, nutrient and energy intake of an individual may not be sufficient. While food frequency questionnaires (FFQs) are widely used to assess dietary intakes in nutritional research due to their convenience, cost-effectiveness and reduced respondent burden, FFQs are prone to recall bias and may provide inaccurate estimations of total energy and nutrients [66]. This could lead to misclassification in the measurement of FV consumption. Therefore, FFQs should be used with caution.

## Conclusion

One-third of our Czech study population was at risk of food insecurity during the late COVID-19 pandemic in 2022, and our study underscores food insecurity and limited consumption of FV as critical social determinants of health, particularly affecting socially and economically vulnerable populations, i.e., younger adults, individuals living in poverty, those with lower educational attainment, limited mobility and poor health. Interventions should go beyond alleviating hunger and focus on promoting healthy eating and sustained access to healthy food. Improving access to healthy food can be achieved through various measures, including financial incentives for purchasing fresh produce, enhancing nutrition education and allocating land for personal or community allotments. A comprehensive, multi-sectoral approach is essential to ensure that food-insecure individuals have both the means and the knowledge to have a healthy diet.

## Data Availability

The datasets used and/or analysed during the current study are available from the corresponding author upon reasonable request.
